# Computational analysis of the tryptophan cation radical energetics in peroxidase Compound I

**DOI:** 10.1007/s00775-022-01925-8

**Published:** 2022-01-21

**Authors:** Thomas L. Poulos, Jenny S. Kim, Vidhi C. Murarka

**Affiliations:** Departments of Molecular Biology and Biochemistry, Chemistry, and Pharmaceutical Sciences, University of Calif, Irvine, Irvine, CA 92697-3900 USA

**Keywords:** Heme peroxidases, Computational biology, Crystallography

## Abstract

Three well-characterized heme peroxidases (cytochrome c peroxidase = CCP, ascorbate peroxidase = APX, and *Leishmania major* peroxidase = LMP) all have a Trp residue tucked under the heme stacked against the proximal His heme ligand. The reaction of peroxidases with H_2_O_2_ to give Compound I results in the oxidation of this Trp to a cationic radical in CCP and LMP but not in APX. Considerable experimental data indicate that the local electrostatic environment controls whether this Trp or the porphyrin is oxidized in Compound I. Attempts have been made to place the differences between these peroxidases on a quantitative basis using computational methods. These efforts have been somewhat limited by the approximations required owing to the computational cost of using fully solvated atomistic models with well-developed forcefields. This now has changed with available GPU computing power and the associated development of software. Here we employ thermodynamic integration and multistate Bennett acceptance ratio methods to help fine-tune our understanding on the energetic differences in Trp radical stabilization in all three peroxidases. These results indicate that the local solvent structure near the redox active Trp plays a significant role in stabilization of the cationic Trp radical.

**Graphical abstract:**

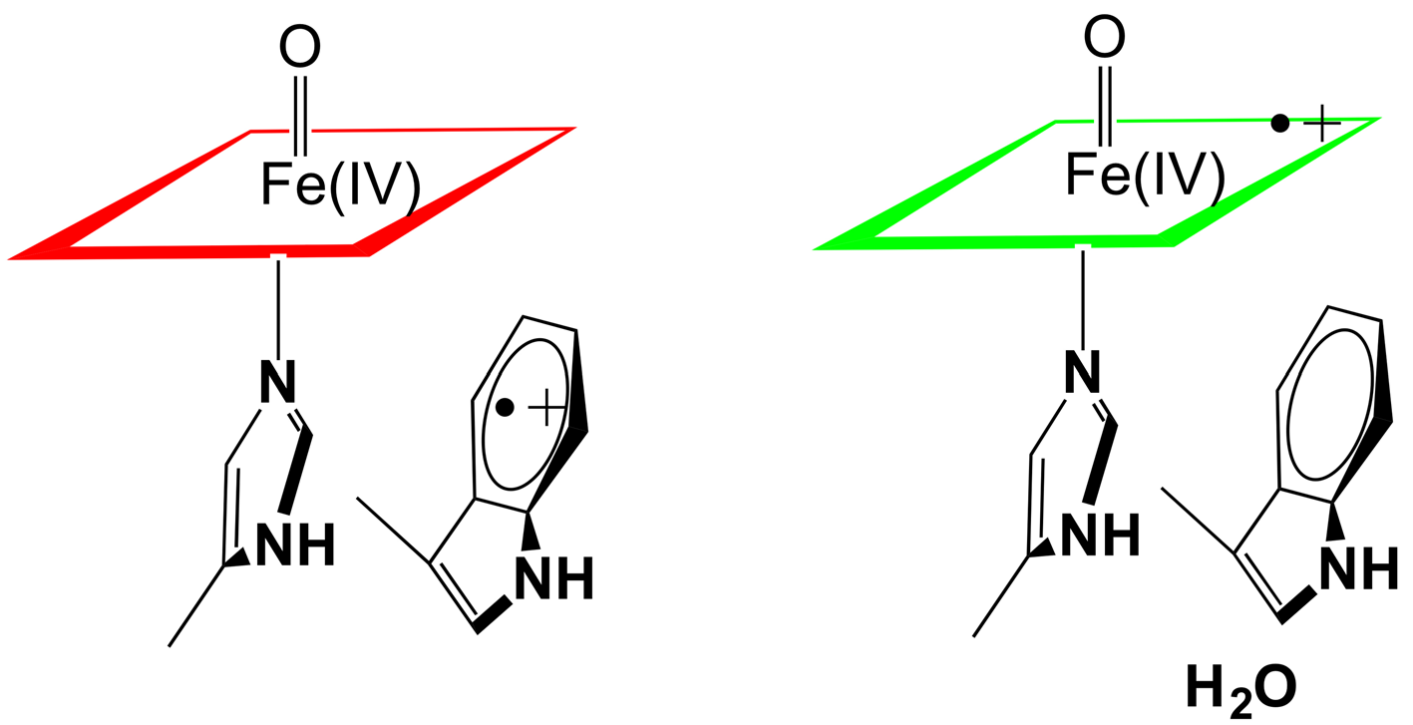

## Introduction

**Fig. 1:**
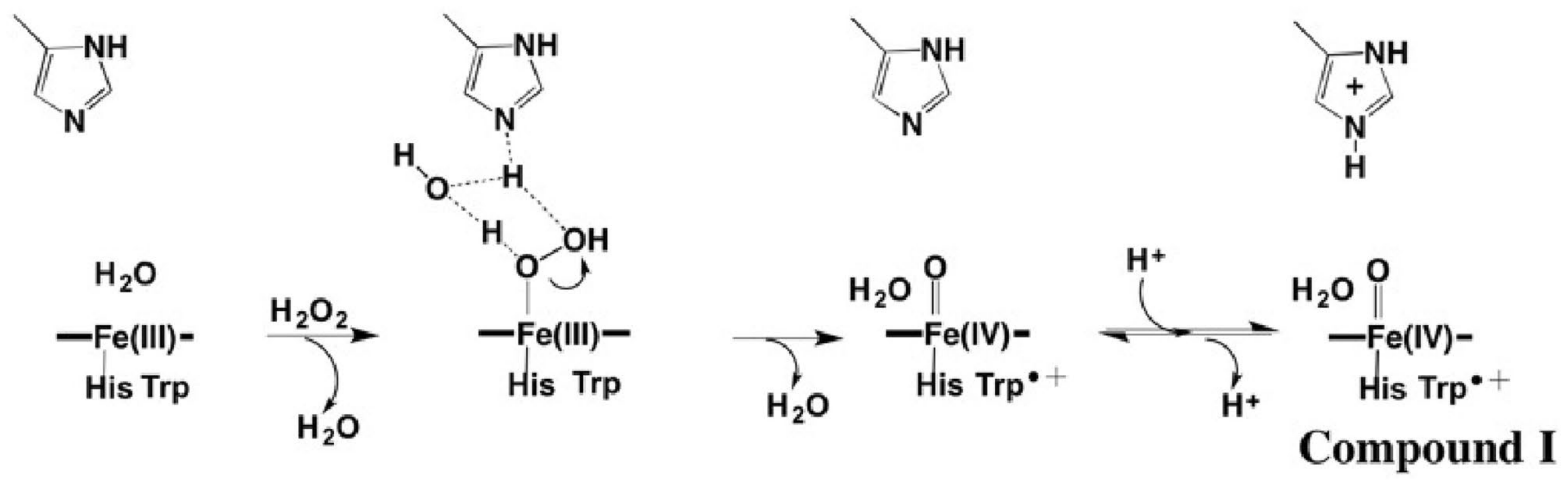
CCP mechanism [1, 2]. Heterolysis of the peroxide O–O bond results in the departure of H_2_O leaving behind an O atom bound to the heme iron. One electron is removed from the heme iron and one from the redox active Trp to give Compound I

Heme peroxidases like cytochrome c peroxidase (CCP) and horseradish peroxidase (HRP) are monomeric proteins consisting of ≈ 300 amino acids and a single heme coordinated to a His residue. The function of peroxidases is to use H_2_O_2_ oxidizing equivalents to oxidize either small organic molecules, as is the case with HRP, or cytochrome c, as with CCP. This is achieved by storing both H_2_O_2_ oxidizing equivalents in the active site (Fig. 1).

One oxidizing equivalent is stored as Fe(IV)=O while the other as an organic radical. With HRP the radical resides on the porphyrin [3] while in CCP the radical is stored on Trp191 which is directly adjacent to the His heme ligand [4, 5]. In HRP the residue corresponding to Trp191 in CCP is a Phe, and it was initially postulated that the reason CCP forms a Trp cationic radical rather than a porphyrin radical is that Trp is simply easier to oxidize than Phe [6]. However, the crystal structure of the closely related ascorbate peroxidase (APX) [7] showed that APX has the same active site Trp as CCP yet APX forms a porphyrin radical [8]. APX, like HRP, has a cation bound about 8 Å from the active site Trp while CCP has no cation leading to the suggestion that the additional nearby positive charge destabilizes a cationic Trp radical [8]. Indeed, engineering the APX cation site into CCP destabilizes the Trp191 cationic radical [9–11]. The question seemed to be settled until the *Leishmania major* cytochrome c peroxidase (LMP) was characterized. The crystal structure [12] shows that LMP has the same active site Trp as CCP, the same cation site as APX, yet LMP forms a very stable Trp radical [13]. LMP differs from both APX and CCP in having a Cys residue very near the active site Trp and mutating this Cys to the corresponding residue in CCP, Thr, destabilizes the Trp radical [13].

Computational methods have been quite helpful in understanding how the local electrostatic environment modulates the redox potential of the active site Trp in peroxidases. The protein dipole Langevin dipoles (PDLD) has provided important insights into Trp radical stabilization [14] with the advantage of computational efficiency relative to free energy perturbation methods and fully solvated atomistic models. Advances in GPU computing, however, provides an opportunity to reexamine the question of Trp radical stability using fully solvated simulations. Here we apply full atomistic molecular dynamics simulations coupled with thermodynamic integration (TI) and free energy perturbation (MBAR = multistate Bennett acceptance ratio) [15] methods to provide an energetic picture on how the local protein environment controls stability of the Trp cationic radical. In addition, we have solved the crystal structure of an APX triple mutant that has previously been shown to enhance Trp radical stabilization.

## Methods

### Computational methods

Crystal structures used were CCP Compound I [16] (3M23) and the resting state for APX (1APX) [7], LMP (3RIV) [12], and CCP engineered to contain the potassium binding site found in LMP and APX [6] (1DJR). The main difference between the resting state and Compound I structure is the presence of the ferryl O atom coordinated to the heme iron and a movement of the distal side active site Arg that H-bonds with the ferryl O atom. The ferryl O atom and position of the Arg in CCP Compound I were modeled into APX and LMP and the CCP potassium site mutant. Other mutants used in the calculations were generated by mutating the side chain of interest in Pymol.

Ferryl heme parameters were from Harris and Loew [22] and the charge parameters used for the Trp cationic radical were from Jensen et al. [14]. The protein together with crystallographic waters were immersed in an octahedral shell of water with a 10 Å cushion and neutralized with sodium ions. Each structure was energy minimized for 1000 cycles while restraining protein heavy atoms followed by 10,000 cycles of minimization with no restraints. The GPU optimized pmed.cuda program [17–19] in Amber 18 or 20 was used for molecular dynamics (MD), thermodynamic (TI) and multistate Bennett acceptance ratio (MBAR) calculations using softcore potential [20, 21]. In these calculations the charge on the active site Trp is changed from 0 (Trp0) to the + 1 radical (Trp+) in 21 λ steps while restraining protein heavy atoms by 10 kcal/mol/Å^2^. Each λ step ran for 2 ns and 5000 data points were output for further processing. One simulation for CCP was carried out for 6 ns with no restraints to test the effect of restraining protein heavy atoms. For the TI method the total ∆G in going from Trp0 to Trp+ is obtained by integrating the change in energy over all λ steps. While TI computes free energy states independently for each λ, MBAR provides a statistically rigorous approach to computing energy differences using multiple states. For example, at configuration λ = i, the energy changes for all 20 λs is tracked. Agreement between these two quite different methods indicates that the computed ∆Gs are giving functionally important information. The last 1 ns of the run was analyzed with the alchemical_analysis python tool [23] that can be downloaded from GitHub (https://github.com/MobleyLab/alchemical-analysis). We also carried out conventional 100 ns MD runs with no restraints to better assess water accessibility to the redox active Trp. All simulations were carried out using GPU clusters at the UCSD Supercomputer Center while post processing to obtain TI and MBAR results was carried out on local workstations.

### Crystal structure

The one missing structure for our computational studies is the mutant of APX that contains the three Met residues found in CCP that are thought to help stabilize the Trp191 radical. To generate the CCP mimic the S160M/L203M/Q204M mutant of soybean APX, a synthetic gene encoding the triple mutant was codon optimized for expression in *E. coli* (GeneScript, Inc.). This mutant is called APX3M. The APX3M gene was subcloned into pCWori (XBal and Ndel). Mutant plasmids were transformed into *E. coli* C41(DE3) cells. The APX3M transformants were grown in 2XYT with ampicillin at 30 °C. The bacteria were grown to an OD_600_ of 0.8–1.0 and induced with 1 mM isopropyl 1-thio-d-galactopyranoside. Upon induction, the temperature was reduced to 25 °C. The cells were incubated for 40–42 h before they were harvested by centrifugation. Cell pellets were resuspended in lysis buffer containing 50 mM Tris (pH 6.4) and lysed through sonication.

After centrifugation, ammonium sulfate was added to reach 40% saturation, and the solution was centrifuged at 15,000 rpm for 1 h at 4 °C. After dialysis to remove the ammonium sulfate, the supernatant was loaded on a DEAE column, and the column was washed with 50 mM Tris (pH 6.4). A gradient of 0–300 mM KCl in the same buffer was used to elute the protein. The protein fractions were pooled, concentrated, and dialyzed overnight against 20 mM KP_i_ (pH 7.4).

The protein then was reconstituted with a fivefold excess of hemin over the course of 1 h, and the protein was loaded onto a DE52 column for removal of the excess heme. Holo-APX3M was eluted with 100 mM KP_i_ (pH 7.4). The eluted protein was dialyzed against 20 mM KP_i_ (pH 7.4) and concentrated. Purity was spectrophotometrically examined, yielding final protein with an *R*_*z*_ 1.9, and was confirmed by SDS-PAGE.

The hanging drop vapor diffusion method was used to grow APX3M crystals. The reservoir solution contained 2.75 M MgSO_4_ and 100 mM Tris (pH 8). Two microliter drops of 10 mg/mL APX3M were mixed with 2 μL drops of the reservoir solution and equilibrated against 700 μL of the reservoir solution at room temperature. Crystals were flash-frozen using liquid nitrogen with Paratone^®^N (Hampton Research) as a cryoprotectant. Data were obtained at the Stanford Synchrotron Radiation Lab on beamline 12–2 and processed with XDS [24]. Initial phases were obtained using the wild type soybean APX structure (1OAG) [25]. The structure was refined to 1.4 Å using Phenix [26]. Refinement statistics are provided in Table 1.

**Table 1:** Crystallographic data collection and refinement statistics

PDB ID	7S10
Resolution range (Å)	34.27–1.4 (1.45–1.4)
Space group	P 4_2_ 2_1_ 2
Unit cell	82.6445 82.6445 75.322 90 90 90
Total reflections	633,979 (23,529)
Unique reflections	51,578 (4861)
Multiplicity	12.3 (4.8)
Completeness (%)	99.38 (95.16)
Mean I/sigma(I)	41.24 (1.48)
Wilson B-factor	17.96
R-merge	0.11 (0.5742)
R-meas	0.1141 (0.6426)
R-pim	0.02982 (0.2727)
CC1/2	0.997 (0.805)
CC*	0.999 (0.945)
Reflections used in refinement	51,560 (4860)
Reflections used for R-free	1701 (161)
R-work	0.1715 (0.2520)
R-free	0.2069 (0.3139)
CC (work)	0.968 (0.878)
CC (free)	0.959 (0.806)
Number of non-hydrogen atoms	2353
Macromolecules	1951
Ligands	54
Solvent	348
Protein residues	249
RMS (bonds)	0.005
RMS (angles)	0.85
Ramachandran favored (%)	98.37
Ramachandran allowed (%)	1.63
Ramachandran outliers (%)	0.00
Rotamer outliers (%)	1.96
Clashscore	3.78
Average B-factor	26.13
Macromolecules	23.94
Ligands	28.20
Solvent	38.06

Numbers in () are the highest resolution shell

## Results

### Crystal structure

**Fig. 2:**
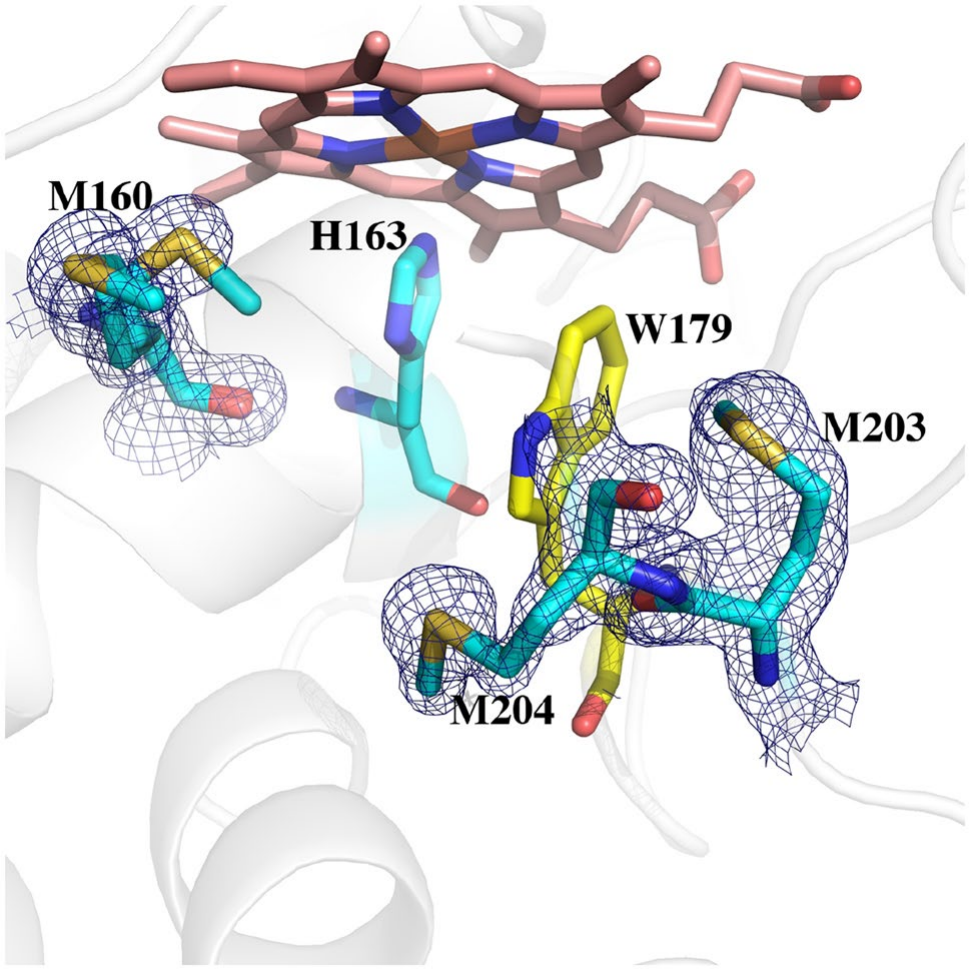
2Fo–Fc electron density map contoured at 1.2σ around the site of the three mutations in APX3M. Met160 is modeled in two conformations. Met204 points into the active site and blocks access of solvent to Trp179 while in wild type APX, Gln204 extends out toward the surface thereby enabling solvent access to Trp179

**Fig. 3:**
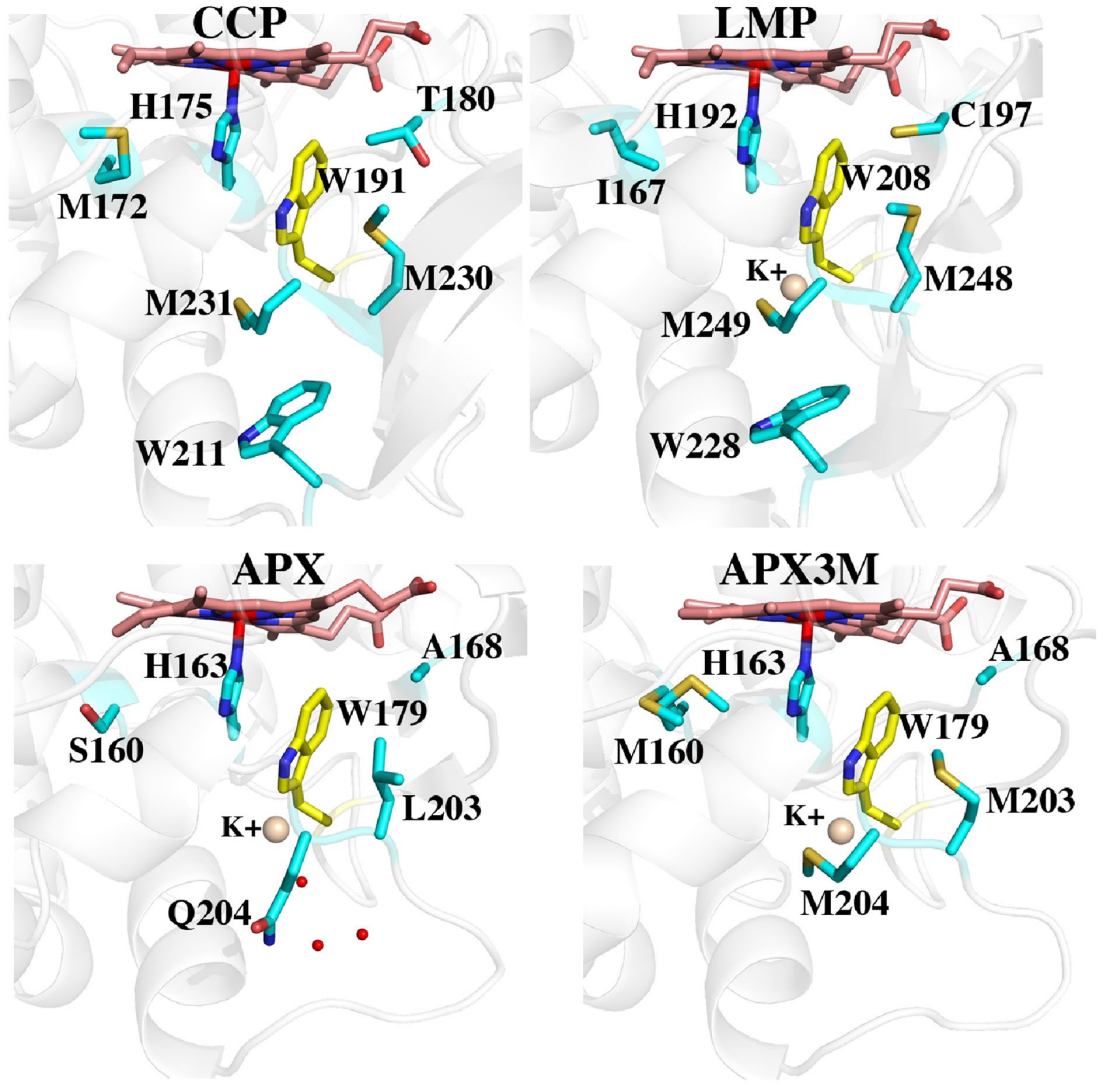
A comparison of all three peroxidases around the redox active Trp (yellow). The Met side chains in APX3M are oriented the same as in CCP and LMP. In wild type APX, Gln204 extends out toward the surface which enables solvent (small red spheres) to access Trp179. The architecture around Trp211 in CCP and Trp228 in LMP helps to seal off the redox active Trp from solvent

All our previous work on APX was with pea cytosolic APX but routine crystallization of mutants proved challenging. Soybean APX, however, crystallizes more easily [25]. Pea and soybean APX share 95% sequence identity with identical active sites so we used the soybean APX3M structure to model the mutations into pea APX. The main reason we solved the APX3M structure rather than simply model in the mutations is the uncertainty of how to position the Q204M mutant side chain. In APX, Gln204 extends out toward the surface while in CCP and LMP the Met points in toward the interior and helps to seal off the redox active Trp from solvent. Our initial computational studies showed that the results changed significantly depending on how Met204 is positioned which provided the motivation for solving the APX3M structure. As shown in Figs. 2 and 3, the three engineered Met residues in APX3M are positioned just as they are in CCP and LMP and we made the safe assumption that these three Met residues will be positioned the same in pea APX.

### Computational results

Figure 3 shows the structures of all three peroxidases plus APX3M in the region surrounding the redox active Trp. Several studies support the view that CCP is better able to stabilize the positive charge on the cationic Trp radical [27–30]. One major factor is the K^+^ site in APX which initially was postulated to destabilize a Trp cationic radical in APX [8]. Some of the strongest evidence stems from engineering studies where introducing the APX K^+^ site into CCP nearly eliminates enzyme activity and greatly destabilizes the Trp191 cationic radical [6]. LMP also has the APX K^+^ yet LMP forms a stable Trp208 radical. The only engineering effort to decipher how LMP might stabilize the positive charge on the Trp radical was to convert Cys203 which contacts Trp208 to the corresponding residue in CCP, Thr. The Cys203Thr mutant loses activity and the Trp208 EPR radical signal is nearly eliminated [12]. To better understand the energetic basis for these differences, Table 2 summarizes the TI/MBAR results where the ∆G in converting neutral Trp0 to Trp+ in Compound I is calculated.

**Table 2:** ∆G of converting neutral Trp0 to cationic Trp+ radical in Compound I of three peroxidases plus various mutants

		TI kcal/mol	MBAR kcal/mol	Comments
0	TRP	− 26.98 (0.038)	− 26.60 (0.067)	Tryptophan alone in solution
1	CCP WT	− 44.19 (0.06)	− 43.89 (0.09)	Crystal structure 3M23
2	CCP WT 6 ns	− 45.76 (0.17)	− 43.63 (0.20)	6 ns unrestrained run
3	CCP K+	− 30.37 (0.03)	− 30.24 (0.05)	Crystal structure of CCP engineered to have APX K^+^ site 1DJR
4	CCP M230L	− 37.30 (0.036)	− 36.36 (0.06)	Based on 1STQ (triple Met mutant with engineered K^+^ site)
5	CCP M231Q	− 38.533 (0.039)	− 37.97 (0.07)	Based on 1STQ. (triple Met mutant with engineered K^+^ site)
6	CCP M230L/M231Q	− 21.84 (0.04)	− 20.45 (0.07)	Based on 1STQ (triple Met mutant with engineered K^+^ site)
7	APX WT	− 27.13 (0.03)	− 27.77 (0.05)	Crystal structure 1APX
8	APX K0	− 39.04 (0.05)	− 39.80 (0.05)	Charge on K^+^ set to zero
9	APX waters	− 30.89 (0.03)	− 31.65 (0.04)	Charges on three waters near active site Trp set to zero
10	APX Q204M	− 32.17 (0.06)	− 33.26 (0.07)	Modeled from APX3M crystal structure
11	APX S160M/L203M/Q204M	− 33.04 (0.03)	− 33.05 (0.04)	APX3M crystal structure
12	LMP WT	− 38.80 (0.04)	− 38.73 (0.06)	Crystal structure 3RIV
13	LMP K0	− 48.43 (0.04)	− 48.37 (0.06)	K^+^ charge set to zero
14	LMP C197T	− 35.12 (0.03)	− 35.91 (0.05)	Crystal structure 3RIW

With the exception of one run, calculations are derived from 2 ns MD simulations where protein heavy atoms were restrained by 10 kcal/mol/Å^2^ and the last 1 ns (2500 data points) used for TI and MBAR calculations. Numbers in () are standard deviations

**Fig. 4:**
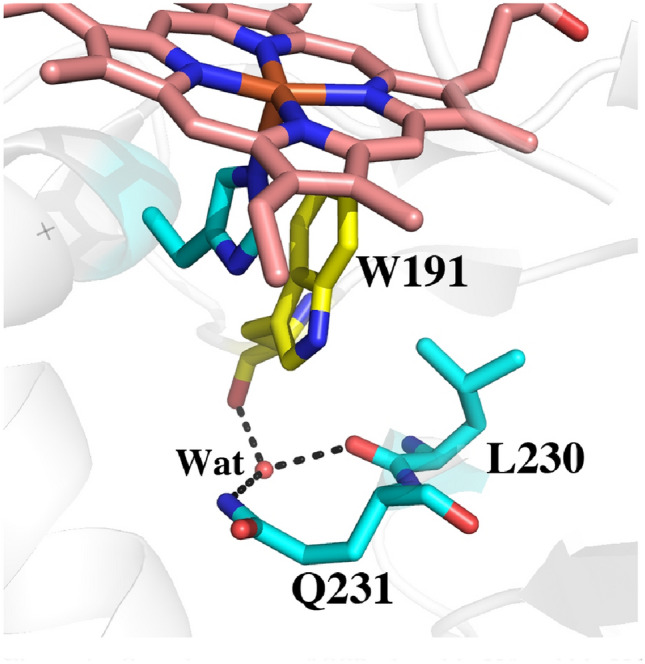
Crystal structure of CCP where Met230 and Met231 were mutated to the corresponding residues in APX (1STQ) [33]. Gln231 points “in” as does Met231 in wild type CCP. However, there now is an internal highly ordered water molecule forming H-bonds with the side chain of Gln231 and the carbonyl O atoms of Trp191 and Leu230

To provide a baseline for comparing relative ∆G values we first computed TI/MBAR for Trp alone in solution using exactly the same protocol and charges used for the protein simulations. As expected CCP (Table 2, rows 1, 2) provides substantial stability to the Trp radical cation relative to free in solution. If we assume that the additional stability of the Trp cationic radical in CCP directly correlates with a change in redox potential, then the TI/MBAR calculations suggest that CCP lowers the redox potential of Trp from 1.1 V free in water [31] to ≈ 0.68 V in CCP. This compares reasonably well with the experimental estimate of ≤ 0.740 V using voltammetry [32] and previous Poisson–Boltzmann electrostatic estimates of ≈ 0.76 V [29]. Also as expected, APX (Table 2, row 7) exhibits reduced stability close to that of Trp alone in water consistent with APX forming a porphyrin cation radical rather than a Trp radical [8]. Engineering the APX K^+^ site into CCP (Table 2, row 3) decreases stability consistent with the loss in activity and substantially reduced EPR signal associated with the Trp191 radical [6, 9, 11]. These results parallel previous computational efforts using the PDLD model [14, 33]. Another important structural feature controlling Trp cation radical stability are two neighboring Met residues in CCP and LMP, Met 230 and Met231 in CCP (Fig. 3). Mutagenesis studies showed that these residues are quite important in stabilizing the Trp191 cationic radical EPR signal [33, 34]. As shown in Table 2 (rows 4, 5), the individual mutations decrease stability about the same ≈ 7–8 kcal/mol. The double mutant (Table 2, row 6), however, where both Met230 and Met231 are mutated to the corresponding residues in APX, Leu and Gln, respectively, exhibit a major decrease in stability. While the addition of these Met residues into APX increases stability of the cationic Trp radical the effect is much less dramatic (Table 2, rows 7, 10, 11). APX, of course, retains the K^+^ site which indicates that the Met residues cannot overcome the destabilizing effects of the K^+^ ion. It should be cautioned that comparisons between APX and CCP have some limitations owing to important structural differences around the site of mutations. In particular is the M231Q mutant in CCP and the corresponding Q204M mutant in APX. As shown in Fig. 3, Gln204 in APX extends out toward the surface of the protein while Met231 in CCP is oriented in where the Met231 side chain approaches close to Trp191. As shown in Fig. 2, the Q204M mutant in APX adopts this same “in” position as in CCP. However, the reverse is not true in CCP since the Gln231 side chain in the M231Q mutant adopts the same “in” orientation similar to the wild type Met231 side chain (Fig. 4). However, an internal water molecule H-bonds with the mutant Gln204 and carbonyl O atoms of Trp191 and Leu230. This is a significant difference and illustrates the limitation of simple computer modeling of mutation sites which would not have accounted for the internal water and the local H-bonding pattern. As we will describe in our discussion of APX, access of solvent to the redox active Trp can have a substantial destabilizing effect which appears to be the case in the CCP double Met mutant.

LMP has the APX K^+^ site but even so Trp cationic radical stability is substantially greater than in APX but less that CCP (Table 2, row 11). Setting the K^+^ charge to 0 in LMP (Table 2, row 13) increases stability ≈ 10 kcal/mol which is close to the same effect as in APX, ≈ 12 kcal/mol (Table 2, row 8). A unique feature of LMP is Cys197 which is near the redox active Trp (Fig. 3). The C197T mutant exhibits much lower activity with a substantially reduced Trp radical EPR signal [12]. The TI/MBAR calculations indicate that this mutation has a fairly modest effect on stability (Table 2, rows 12, 14), ≈ 3.8 kcal/mol. This, of course, assumes that Cys197 remains fully protonated in Compound I although the pKa of Cys197 estimated with the H++ server (http://biophysics.cs.vt.edu/) and constant pH calculations both indicate that Cys197 is protonated at neutral pH. The effect of Cys197 could well be beyond simple electrostatic stabilization. The Cys sulfur is only 3.6 Å from planar face of the redox active Trp indole ring. Such a close interaction with the indole pi electrons could potentially provide greater stabilization to a delocalized radical. Indeed, the shape of the EPR Trp radical signal in LMP is quite different than in CCP [12] which very likely is due to differences in how the unpaired spin interacts with the local environment. Deciphering if Cys197 contributes more than simple electrostatic stabilization will require a much deeper analysis of the LMP Compound I radical spectral properties.

**Fig. 5:**
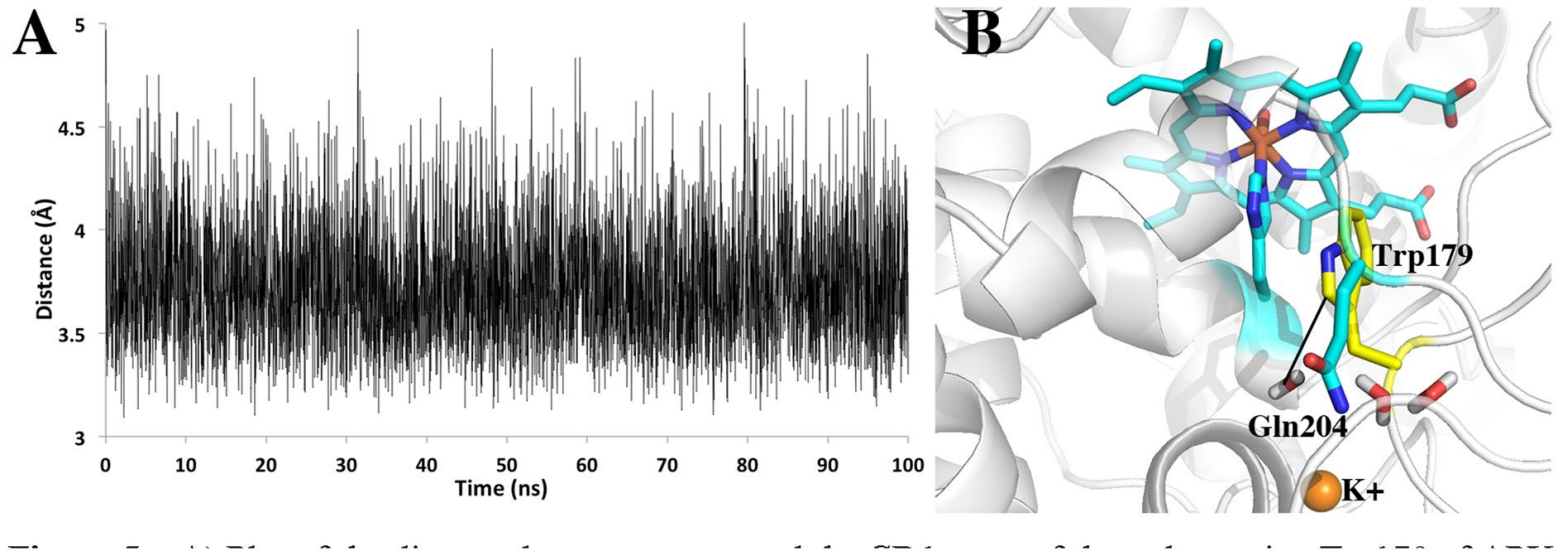
**A** Plot of the distance between water and the CD1 atom of the redox active Trp179 of APX over the course of a 100 ns MD simulation. **B** The line between the water and Trp179 is the distance followed in the panel A plot. The water close to Trp179 is part of a necklace of water molecules that connects the internal water near Trp179 to bulk solvent

One final significant difference between APX and LMP/CCP that cannot be tested by simple mutagenesis is the solvent accessibility of the redox active Trp. As shown in Fig. 3, APX lacks the local structure around the Trp211 region of CCP. This lack of local restrictions allows Gln204 in APX to extend out in solution while the CCP M231Q mutant must still point in toward the redox active Trp191. As a result, the redox active Trp in APX is more accessible to water. To better understand the dynamics of solvent access we carried out an unrestrained 100 ns MD simulation of wild type APX and saved snapshots every 20 ps for a total of 5000 snapshots. Figure 5A tracks the distance between water and the redox active Trp over the 100 ns MD run. The distance monitored is the line connecting the water and the CD1 atom of Trp179 (Fig. 5B). This distance remains less than 4 Å 83% of the time.

Such close proximity of water to Trp179 could be one reason why Trp179 does not form a stable cationic radical. To test this hypothesis, we mimicked the removal of the water electrostatic effects, if any, by setting the charges of the three water molecules to zero but restraining these waters in position by 10 kcal/mol/Å^2^. Table 2 row 9 shows that neutralizing these waters increases stability of the cationic Trp radical by about − 3.7 kcal/mol. The APX Q240M mutant mimics (Table 2, row 7) this effect by replacing the waters with Met204 which points “in” effectively preventing water from approaching the redox active Trp. The increased stabilization of the Q204M mutant vs simply neutralizing the waters very likely is due to the electronegative Met sulfur atom.

## Conclusions

**Fig. 6:**
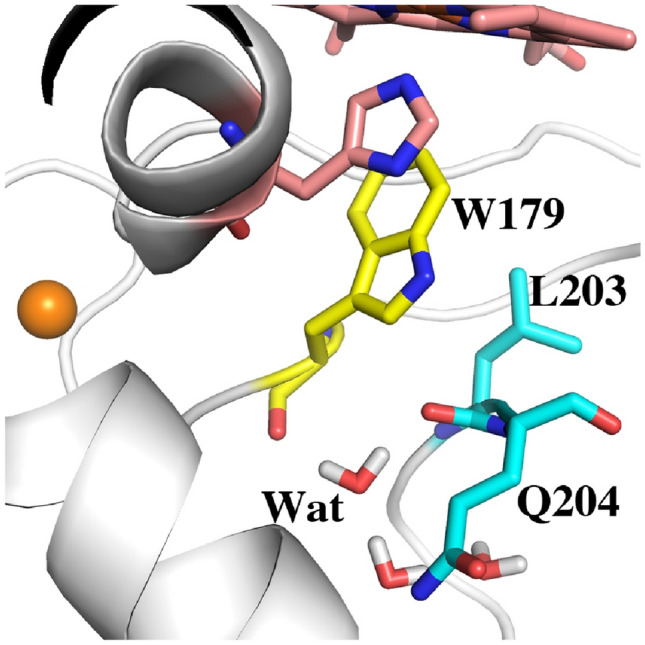
Snapshot from the 100 ns MD simulation of wild type APX Compound I. The water labeled as **Wat** is quite stable and remains H-bonded to the carbonyl O atoms of Trp179 and Gln204. This same water molecule is present in the Met231Gln mutant of CCP (Fig. 4) although in the CCP mutant, the mutant Gln204 side chain points “in” and forms an additional H-bond **Wat**

The present study provides a simplified energetic assessment of how the protein modulates and controls stability of the Trp cationic radical in three peroxidases. Perhaps the most important new insight is the role that neighboring solvent plays in Trp cation radical stability. As shown in Fig. 3, APX lacks the local architecture surrounding Trp211 in CCP (Trp228 in LMP) that helps to seal off the redox active Trp from solvent. In APX Gln204 extends “out” rather than “in” like the Met residues in CCP and LMP. As a result, solvent has more ready access to APX Trp179. Figure 6 shows the location of the water structure unique to APX that is stable over the 100 ns unrestrained MD simulation. Of particular importance is the water molecule labeled as **Wat**. This water donates stable H-bonds to the carbonyl O atoms of Trp179 and Leu204. The same water is found in the CCP mutant designed to mimic APX (Fig. 4). The close proximity of the carbonyl O atoms to Trp179 should help to stabilize a cationic Trp radical. This is the case in all 3 peroxidases but the water present in APX and the CCP APX-mimic mutant with its H-bonding interactions may possibly attenuate the electrostatic stabilization of the carboxyl O atoms. The computational results where the water charges are neutralized or Gln204 is replaced by Met supports this view. In summary, our present study is consistent with previous computational efforts with additional insight on the role solvent molecules can play in cationic Trp radical stability. Moreover, the approach used here is straightforward and requires only a modest level of computational expertise which should help in the use of similar methods to assist in the design and modulation of redox active centers in metalloenzymes.
